# Chromosome-level genome of the Adriatic sturgeon, *Acipenser naccarii*: A resource for polyploid fish genomics

**DOI:** 10.1093/jhered/esag023

**Published:** 2026-03-12

**Authors:** Roberto Biello, Annalisa Scapolatiello, Sebastiano Fava, Victor Hugo Muñoz Mora, Stefano Dalle Palle, Patrícia Santos, Alessio Iannucci, Tatiana Tilley, Nivesh Jain, Jennifer Balacco, Brian O'Toole, Giulio Formenti, Erich D Jarvis, Claudio Ciofi, Emiliano Trucchi, Leonardo Congiu, Giorgio Bertorelle, Andrea Benazzo

**Affiliations:** Department of Life Sciences and Biotechnology, University of Ferrara, Ferrara, Italy; Department of Biology, University of Padua, Padua, Italy; Department of Life and Environmental Sciences, Marche Polytechnic University, Ancona, Italy; Department of Life Sciences and Biotechnology, University of Ferrara, Ferrara, Italy; Department of Biology, University of Padua, Padua, Italy; Department of Life Sciences and Biotechnology, University of Ferrara, Ferrara, Italy; Department of Biology, University of Florence, Florence, Italy; The Vertebrate Genome Laboratory, The Rockefeller University, New York, NY, United States; The Vertebrate Genome Laboratory, The Rockefeller University, New York, NY, United States; The Vertebrate Genome Laboratory, The Rockefeller University, New York, NY, United States; The Vertebrate Genome Laboratory, The Rockefeller University, New York, NY, United States; Department of Biology, University of Florence, Florence, Italy; The Vertebrate Genome Laboratory, The Rockefeller University, New York, NY, United States; The Vertebrate Genome Laboratory, The Rockefeller University, New York, NY, United States; Howard Hughes Medical Institute, Chevy Chase, MD, United States; Department of Biology, University of Florence, Florence, Italy; Department of Life and Environmental Sciences, Marche Polytechnic University, Ancona, Italy; Department of Biology, University of Padua, Padua, Italy; National Biodiversity Future Center, Palermo, Italy; Department of Life Sciences and Biotechnology, University of Ferrara, Ferrara, Italy; Department of Life Sciences and Biotechnology, University of Ferrara, Ferrara, Italy

**Keywords:** conservation genomics, endemic species, endemixit, genome assembly, polyploidy

## Abstract

The Adriatic sturgeon, *Acipenser naccarii*, a tetraploid species endemic to the North Adriatic region, has experienced significant population declines, resulting in its classification as “Critically Endangered” by the International Union for Conservation of Nature (IUCN). Historically widespread in the Adriatic Sea’s tributaries, the species is now at high risk of extinction with occasional reproductions occurring in the wild. Using long-read sequencing (PacBio HiFi) and chromatin conformation capture sequencing (Hi-C), we generated a phased reference genome for the tetraploid Adriatic sturgeon. The haploid assembly spans 1.94 Gb across 2,083 scaffolds, with a contig N50 of 1.024 Mb, a scaffold N50 of 39.6 Mb, and a scaffold L90 of 276. Approximately 80% of the genome is contained within the first 60 scaffolds, indicating a high degree of contiguity. The Benchmarking Universal Single-Copy Orthologs (BUSCO) completeness score of the haploid assembly reached 88.9%, whereas combined metrics for all four haploid assemblies increased to 94.1%. This comprehensive genomic resource provides valuable insights into the genetic and evolutionary mechanisms of polyploidy and, more specifically, it will improve our understanding of the genetic diversity of the Adriatic sturgeon, thereby informing targeted conservation strategies for this critically endangered species.

## Introduction

**Fig. 1:**
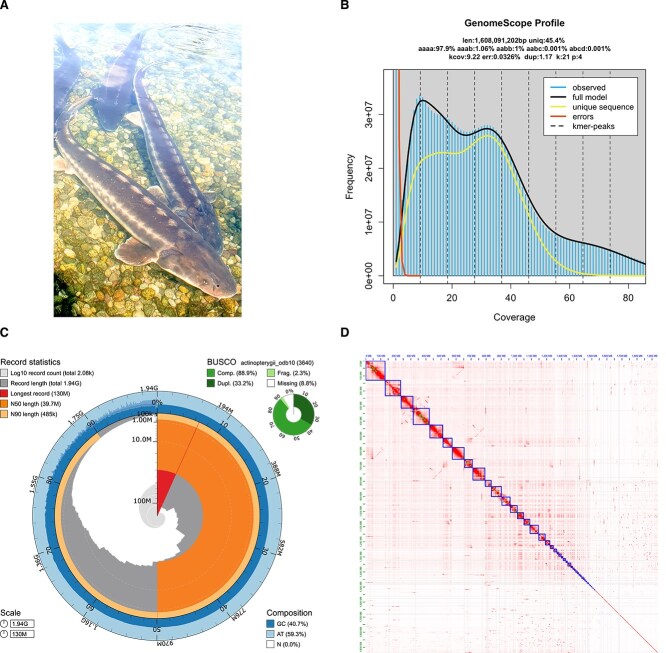
Visual overview of *Acipenser naccarii* genome assembly metrics. a) Photography of *A. naccarii* individuals (photo credit: Annalisa Scapolatiello). b) K-mer spectrum output and corresponding genome size and heterozygosity estimated with GenomeScope 2.0. c) BlobToolKit snail plot showing a graphical representation of the quality metrics presented in Tables 2 and 3 for the *A. naccarii* assembly (hap1). The longest scaffold (red vertical line), N50 (orange track), N90 (light orange track), GC content (external blue track) ,and BUSCO scores (outer circular pie chart in green) are shown. d) Hi-C contact map for the scaffolds of the hap1 genome assembly generated with Juicebox.

The Adriatic sturgeon, *Acipenser naccarii* (Fig. 1a), is a critically endangered tetraploid endemism of the Adriatic region. Once abundant, the species experienced a catastrophic population decline throughout the 20th century, culminating in its near extinction in the wild by the 1990s.

The Adriatic sturgeon’s decline reflects the broader crisis facing sturgeons worldwide. As ancient Acipenseriformes, their late maturity, long lifespans, and size-dependent fecundity once provided evolutionary advantages but now make them highly vulnerable to human impacts (Billard and Lecointre 2000). Many sturgeons are anadromous, requiring access to freshwater spawning grounds like gravel beds for successful reproduction (Sulak and Randall 2002). However, dams and other barriers have disrupted these migrations, cutting off critical habitats (Williot et al. 2002). Overexploitation for caviar and meat has further accelerated declines by disproportionately removing the largest, most reproductively valuable individuals.

Its persistence today is largely attributable to a single founder stock preserved in a private aquaculture facility (Congiu et al. 2011). Over the past four decades, intensive conservation programs, primarily centered in Italy’s Po River basin, have tried to recover the species population by releasing hundreds of thousands of juvenile sturgeons derived from this limited genetic pool (Arlati and Poliakova 2009). However, the species’ long-life cycle, spanning more than 50 years, means that the success of these efforts will only be measurable over extended timescales. Only recently, in fact, occasional natural reproductions have been recorded in the Ticino and Livenza rivers allowing the reclassification by IUCN from *Critically Endangered and Possibly Extinct in the Wild* to *Critically Endangered*.

The ploidy level of sturgeon species has been a subject of scientific debate, primarily due to the complex evolutionary history involving multiple whole-genome duplication events. According to Birstein et al. (1997) and Ludwig et al. (2001), the common ancestor of all sturgeons had a diploid genome with approximately 60 chromosomes. A first major whole-genome duplication likely occurred during the early Jurassic period (Fontana et al. 2008), leading to a tetraploid condition with around 120 chromosomes. Over time, however, this genome underwent a process known as functional diploidization (Stift et al. 2008), during which, despite the doubled chromosome count, the genome, or at least a relevant part of it, began to function more like a diploid. This has led to debate over whether these sturgeons with 120 chromosomes should be considered tetraploid or functionally diploid. Subsequently, further independent duplication events occurred both within the Pacific and Atlantic sturgeon clades. These events gave rise to species with approximately 240 chromosomes. Although this chromosome count would nominally suggest an octoploid genome, they are mostly functionally tetraploid. To further complicate the picture, also following this last duplication events, a functional diploidization process likely started to reduce the genomic redundancy (Stift et al. 2008).

The Adriatic sturgeon is one of the 240 chromosome species and is generally considered as a functional tetraploid, as revealed by in situ hybridization analyses of 28S and 5S rDNA (Fontana et al. 1999) and by microsatellite data, with most loci exhibiting up to four alleles per individual. Nevertheless, some microsatellite loci exhibit more than four alleles in numerous individuals (Boscari et al. 2015) either due to local duplication events or as a legacy of the nominal octoploid condition (Ludwig et al. 2001; Havelka et al. 2013). On the other hand, a functional diploidization is taking place as shown by some microsatellite loci with four alleles per individual that segregate according to a double disomic pattern (Dalle Palle et al. 2022). Consequently, in the genome of *A. naccarii*, as well as in all the 240 chromosome species, it is possible that different genomic regions present different levels of ploidy (diploid, tetraploid, octoploid), though functionally tetraploid regions are expected to predominate.

Here, we present a high-quality chromosome-scale reference genome for *A. naccarii* (Fig. 1a), produced as part of ENDEMIXIT (www.endemixit.com) and Vertebrate Genomes Project (https://www.vertebrategenomesproject.org/). This high-quality reference genome is a valuable resource for both the conservation of this critically endangered species and the study of genome evolution in sturgeons. As one of the first chromosome-level assemblies available for polyploid sturgeons (Wang et al. 2024), it offers key insights into the genetic and evolutionary mechanisms underlying polyploidy, contributing to a broader understanding of genome duplication in vertebrates. In the face of persistent environmental and anthropogenic challenges, this genomic resource represents a critical step toward to help improving the conservation strategy for this species.

## Material and methods

### Sampling, genomic DNA extraction, and sequencing

Two juvenile sturgeon specimens of unknown sex, born as a result of the June 2020 breeding program, were collected after natural death and generously donated by Allevamento Storione Ticino, located in Cassolnovo (Italy). The first was used for genome sequencing, whereas the second specimen was utilized for transcriptome analysis.

All the following steps were carried out at the Vertebrate Genomes Laboratory (https://vertebrategenomelab.org/) of the Rockefeller University. High molecular weight (HMW) DNA was extracted from muscle with the Circulomics HMW DNA extraction standard TissueRuptor protocol with the Nanobind Tissue Big DNA Kit (PN NB-900-701-01). DNA absorbance was checked for quality and purity control with Nanodrop, and average fragment length was verified with a pulsed field gel electrophoresis. Genomic data from 2 different sequencing technologies were used for the assembly: Pacific Biosciences (PacBio) High Fidelity (HiFi) reads and Hi-C reads from Arima Genomics. PacBio HiFi libraries were prepared using the Pacific Biosciences Express Template Prep Kit 2.0. The library was then size selected (>10 kb) using the Circulomics Short Read Eliminator. The PacBio library was sequenced on 2 PacBio 8M v3 SMRT Cells on a PacBio Sequel II and 1 PacBio 8M SMRT Cell on a PacBio Sequel IIe using the sequencing kit 2.0 and a 30-h movie. Hi-C libraries were generated by Arima Genomics (https://arimagenomics.com/) using muscle in vivo cross-linking with the Arima-HiC kit with 2-enzyme proximity ligation. Proximally ligated DNA was subjected to shearing, size selection (~200 to 600 bp) with Solid-Phase Reversible Immobilization (SPRI) beads, and enrichment with streptavidin beads for the biotin-labeled DNA. KAPA Hyper Prep kit was employed to generate libraries compatible with Illumina technologies. Libraries were amplified through Polymerase Chain Reaction (PCR), purified with SPRI beads, and sequenced on an Illumina HiSeq X (~60× coverage) after a quality check with Bioanalyzer and Quantitative PCR (qPCR).

### RNA extraction and sequencing

RNA sequences were obtained from four different tissues: muscle, liver, gonads, and brain. RNA extraction was performed using the Direct-zol RNA Miniprep Kits, following the manufacturer’s instructions. The total extracted RNA was subsequently subjected to RNA sequencing on a NextSeq 500 sequencer (Illumina) at the Next-Generation Sequencing (NGS) facility of the University of Padua, Italy.

### Genome size and ploidy estimation

Quality control of HiFi reads was performed using fastqc v0.11.8 (Andrews 2010; http://www.bioinformatics.babraham.ac.uk/projects/fastqc/). Residual sequencing adapters were identified and removed using HiFiAdapterFilt v2.0.0 (Sim et al. 2022), setting a minimum of 44 bp and a 97% identity with the known adapter sequence.

To estimate the genome size of the Adriatic sturgeon, the KMC v3.0 tool (Kokot et al. 2017) was used to generate a k-mer spectrum with a k-mer length of 21 and filters set to -ci10 and -cx1200 to exclude low-frequency and excessively high-frequency k-mers, respectively. The k-mer spectrum was then analyzed using GenomeScope v2.0 (Ranallo-Benavidez et al. 2020), which uses k-mer frequency distributions to estimate genome size, repetitive content, and heterozygous/homozygous fraction of the genome.

To assess the ploidy of the genome, Smudgeplot v0.3.0 (Ranallo-Benavidez et al. 2020) and nQuire v1.0 (Weiß et al. 2018) were employed. Smudgeplot provides a reference-free approach to analyze genomes with complex ploidy levels by interpreting patterns in k-mer pair frequencies. Instead, nQuire requires a Binary Alignment Map (BAM) file as input. For this reason, the HiFi reads were aligned to the *Acipenser ruthenus* reference genome (Du et al. 2020). nQuire calculates base frequencies at biallelic Single Nucleotide Polymorphism (SNP) sites. These frequencies are compared with three predefined diploidy, triploidy, and tetraploidy models to infer ploidy levels.

### Genome assembly

The *A. naccarii* genome was assembled using the VGP 2.0 assembly pipeline (Rhie et al. 2021), as outlined in Table 1, with an additional bioinformatic step to account for the ploidy of the species during Hi-C scaffolding.

**Table 1:** Pipeline and software used for the genome assembly.

Assembly step	Software	Version	Reference
**Initial assembly**			
Reads quality control	FastQC	0.11.8	Andrews (2010)
Adaptors residual removal	HiFiAdapterFilt	2.0.0	Sim et al. (2022)
k-mer spectrum generation	KMC	3.0	Kokot et al. (2017)
Estimation of genome size and heterozygosity	GenomeScope	2.0	Ranallo-Benavidez et al. (2020)
Estimation of genome ploidy	Smudgeplot	0.3.0	Ranallo-Benavidez et al. (2020)
	nQuire	1.0	Weiß et al. (2018)
**Genome assembly and scaffolding**			
De novo assembly (contigging)	Hifiasm	0.19.3	Cheng et al. (2021a); Cheng et al. (2022)
Hi-C alignment	Chromap	0.2.4	Zhang et al. (2021)
Contact processing	pairtools	1.0.2	Open2C et al. (2024)
Hi-C contact map visualization	Juicebox	2.16	Durand et al. (2016)
Hi-C scaffolding	Yahs	1.2	Zhou et al. (2023)
	RagTag	2.1	Alonge et al. (2022)
**Genome annotation**			
TE annotation	TE Annotator	1.9.9	Ou et al. (2019)
	DeepTE	1.0	Yan et al. (2020)
	RepeatMasker	4.1.2	Smit et al. (2021)
RNA-seq raw reads trimming	fastp	0.19.5	Chen et al. (2018)
Mapping RNA-seq reads genome	HISAT2	2.2.1	Kim et al. (2019)
SAM/BAM processing	samtools	1.11	Li et al. (2009)
Filtering splice junctions	Portcullis	1.2.4	Mapleson et al. (2018)
Gene prediction	Braker	3.0	Gabriel et al. (2024)
Gene combining	TSEBRA	1.0.3	Gabriel et al. (2021)
**Mitochondrial assembly**			
De novo genome assembly	MitoHiFi	3.2.1	Uliano-Silva et al. (2023)
**Genome quality assessment**			
Assembly completeness	BUSCO	5.3.2	Manni et al. (2021)
	Merqury	1.3	Rhie et al. (2020)
**Contamination screening**			
General contamination screening	BlobTools	1.1.1	Laetsch and Blaxter (2017)
**Genome synteny**			
Synteny	GENESPCACE	1.2.3	Lovell et al. (2022)

HiFi and Hi-C reads were jointly processed in Hifiasm v0.19.3 (Cheng et al. 2021a; Cheng et al. 2022) in tetraploid mode (--n-hap 4) to generate 4 phased haploid genome assemblies. Each genome assembly was then processed to identify and remove possible artifacts due to the presence of multiple allelic haplotigs from the same genomic regions, similarly to what was performed in Kuhl et al. (2022). Briefly, Hi-C reads were aligned to each assembly independently using Chromap v0.2.4 (Zhang et al. 2021) and pairtools v1.0.2 (Open2C et al. 2024) was then used to retain paired-end reads having a mapping quality >20. Each scaffold was divided into not-overlapping 50 Kb windows, and the number of Hi-C contacts was counted for each pair of windows based on the alignments. Windows that did not contain any restriction site were excluded from the analysis. The observed frequency distribution of intra-windows contacts was then used to fit a mixture Poisson distribution model and identify the homozygous windows. The noise parameter epsilon, representing the maximum number of spurious contacts between independent regions of the genome, was estimated by selecting the terminal windows of each scaffold in homozygous state and counting the number of contacts between the ones located in different scaffolds. Epsilon was set to the maximum observed value among all the possible window pairs. Each scaffold pair was compared, retaining the scaffold windows showing a number of contacts higher than epsilon. The window positions along the two scaffolds were used to perform a nonparametric Kendall correlation test. In each comparison, the shorter scaffold was classified as an allelic haplotig and removed from the assembly if it showed a proportion of valid windows greater than 0.6 and a significant correlation test result (*P* < 0.01). The four haploid assemblies were independently purged following the above bioinformatic procedure. A further scaffolding round for each haploid assembly was performed with Yahs v1.2 (Zhou et al. 2023) using Hi-C reads, setting the restriction enzyme motif “GATC, GANTC, CTNAG, TTAA” as suggested for Arima Genomics data. Finally, the manual curation of each assembly was performed using Juicebox v2.16 (Durand et al. 2016). To further improve the quality of the assemblies, potential misassemblies were detected in the most contiguous haploid assembly, among the four assembled, using the RagTag v2.1 (Alonge et al. 2022) *correct* module, setting the chromosome-scale *A*. *ruthenus* genome as the reference sequence. The correction step was conducted using the long-reads coverage profile to validate each putative misassembly point, hence a breakpoint was inserted only if supported by the reference alignment and a concurrent drop in sequencing depth. The RagTag *scaffold* module was then used to perform a final scaffolding round on the corrected haploid assembly, introducing the proposed joints only if they resulted in an improved Hi-C contact pattern by a visual inspection. The resulting curated haploid assembly was finally used as a guide (reference sequence) to correct and scaffold the other three haploid assemblies using RagTag. Also in this step, the proposed edits from RagTag were introduced in the assemblies if compatible with the observed Hi-C contact matrix using the Juicebox platform.

We assessed the quality of our genome assemblies using three methods. First, we used the BUSCO quality control tool to check for genome completeness using a set of conserved single-copy orthologous genes. We ran BUSCO v5.3.2 (Manni et al. 2021) in the genome mode with default parameters on the Actinopterygii dataset (Actinopterygii_odb10) that contains 3,640 orthologous genes. Second, we used Merqury v1.3 (Rhie et al. 2020) to estimate base-level accuracy, expressed as the phred-scaled Quality Value (QV) and assembly completeness by comparing the k-mers in the assembly with those observed in the HiFi reads. Third, we used the blobtools pipeline v1.1.1 (Laetsch and Blaxter 2017) by generating taxon-annotated Guanine-Cytosine (GC) content-coverage plots (known as “BlobPlots”). Each scaffold was annotated with taxonomy information based on blastn (Basic Local Alignment Search Tool) v2.12.0 (Camacho et al. 2009) searches against the National Center for Biotechnology Information nucleotide database with the options “-outfmt ‘6 qseqid staxids bitscore std sscinames sskingdoms stitle’ -culling_limit 5 -evalue 1e-25.” To calculate average coverage per scaffold, we aligned the PacBio HiFi raw reads to the assembly using bwa-mem version 0.7.17 (Burrow-Wheeler Aligner) (Li et al. 2009) with default parameters. The resulting BAM file was sorted with samtools v1.11 (Li et al. 2009) and passed to blobtools along with the table of blastn results.

### Mitochondrial genome assembly

The mitochondrial genome was assembled and annotated with MitoHiFi v3.2.1 (Uliano-Silva et al. 2023), using a genome from *A. naccarii* (MK078265.1; Cheng et al. 2021b) as a reference.

### Genome annotation

To identify and annotate repetitive elements, we first generated a de novo repeat library using the Extensive de novo TE Annotator v1.9.9 (Ou et al. 2019). Subsequently, we refined the library using DeepTE v1.0 (Yan et al. 2020), which employs convolutional neural networks to classify unknown elements at the order and superfamily levels. Then, we used RepeatMasker v4.1.2 (Smit et al. 2021; http://www.repeatmasker.org) with the final library to mask the genome and parsed the RepeatMasker output file with RM_TRIPS script (https://github.com/clbutler/RM_TRIPS). Transposable element landscapes were generated using the RepeatMasker script calcDivergenceFromAlign.pl.

Quality control and trimming for adapters and low-quality bases (quality score <20) of the RNA-seq raw reads were performed using fastqc v0.11.8 (Andrews 2010) and fastp v0.19.5 (Chen et al. 2018), respectively. After trimming, reads shorter than 75 nucleotides were discarded. High-quality reads were then mapped to the soft-masked assembly with HISAT2 v2.2.1 (Kim et al. 2019) and sorted with samtools v1.11 (Li et al. 2009). All the BAM files were filtered to remove invalid splice junctions with Portcullis v1.2.4 (Mapleson et al. 2018). Filtered RNA-seq alignments were passed to Braker v3.0 (Gabriel et al. 2024), together with protein sequences of three closely related species, *Acipenser oxyrinchus* (GCA_030684275.1), *A*. *ruthenus* (GCF_902713425.1), and *Polyodon spathula* (GCF_017654505.1). The Braker gene prediction pipeline was run with the options “—softmasking.” This pipeline uses StringTie (Pertea et al. 2015) to assemble the RNA-seq reads followed by rounds of GeneMark and AUGUSTUS training and gene prediction (Stanke and Waack 2003). Gene sets were combined with TSEBRA v1.0.3 (Gabriel et al. 2021). The completeness of the final gene set was checked with BUSCO v5.3.2 (Manni et al. 2021) against the Actinopterygii dataset (Actinopterygii_odb10), using the longest transcript of each gene as the representative transcript.

### Synteny analysis

Syntenic blocks of genes in *A. naccarii* were identified across the four haploid assemblies and compared with *A*. *ruthenus* (GCF_902713425.1), *A. oxyrinchus* (GCA_030684275.1), and *Huso huso* (GCA_036884735.1), using GENESPACE v1.2.3 (Lovell et al. 2022). As outgroup, the genome of *P. spathula* (GCF_017654505.1) was used. Riparian plots were obtained using the synteny calculation blocks obtained from GENESPACE.

## Results and discussion

### Genome size and ploidy estimation

GenomeScope reported a haploid genome size of ~1.6 Gbp for *A. naccarii*, which is 11 % to 20% smaller than those reported for other sturgeon species, such as *Acipenser gueldenstaedtii* (1.9 Gbp), *A*. *ruthenus* (1.8 Gbp), *Acipenser baerii* (2.0 Gbp), and *Acipenser sinensis* (1.9 Gbp) (Zhou et al. 2011; Du et al. 2020; Wang et al. 2024) (Fig. 1b). The reported homozygosity percentage was 97.9% (*aaaa*), and the most frequent types of heterozygous alleles were *aaab* at 1.06% and *aabb* at 1% (Fig. 1c). Typically, autotetraploid genomes have *aaab* > *aabb*, whereas allotetraploid genomes have the opposite (*aaab* < *aabb*). In the case of the Adriatic sturgeon, there was not a substantial difference between *aaab* and *aabb*, which does not allow to assess if the two events of whole-genome duplication in this species (Vasil’eva 1999; Fontana et al. 2008) occurred through auto- or allopolyploidy duplications. The Smudgeplot analysis identified *aabb* (35%) and *aaab* (12%) genotype proportions, suggesting a predominantly tetraploid genome structure in the Adriatic sturgeon. The remaining 53% of the genome was fractionated in higher ploidy levels that could not be clearly resolved (Supplementary Fig. S1). This pattern may reflect technical limitations of the tool due to the high repetitiveness of the genome (54.6%), a level well above the condition under which Smudgeplot reliably infers ploidy in autotetraploid datasets (<38% repetitiveness; Ranallo-Benavidez et al. 2020). Under such complexity, k-mer based approaches may overestimate ploidy because the signal from repetitive k-mers exceeds that from heterozygous k-mers. However, considering the evolutionary history of the Adriatic sturgeon, which likely underwent two whole-genome duplications (Fontana et al. 2001; Biltueva et al. 2020), it could be considered octoploid on an evolutionary scale. Therefore, the smudges above tetraploidy observed in Supplementary Fig. S1 should be interpreted with caution as they might reflect remnants of the historical octoploid state. To further investigate the polyploid structure of the *A. naccarii* genome and possibly overcome the Smudgeplot’s extremely high-coverage requirements, we employed the read mapping approach implemented in nQuire. nQuire was applied to each of the 60 chromosomes individually. Thirty-five chromosomes fit the tetraploid model, whereas five matched the diploid model (Supplementary Fig. S2). The remaining 20 chromosomes did not fit any model, likely due to a low presence of biallelic sites or potential octoploidy, as suggested by Smudgeplot. However, nQuire does not support an octoploid model. Despite differences in the estimated proportion of tetraploid regions (74.7% in nQuire vs. 47% in Smudgeplot), both methods indicated that the Adriatic sturgeon genome is predominantly tetraploid. These results provided a first insight into the complex ploidy levels present in the genome of this species.

### Genome assembly

We generated 5.96 million HiFi reads and 357 million of Hi-C read pairs, corresponding to an approximate sequencing coverage of 59X and 67X, respectively. The assembly process resulted in four haploid assemblies having a genome size between 1.65 and 1.94 Gbp, a N50 from 37.8 to 56.8 Mb, and L50 from 11 to 33 scaffolds (Fig. 1c; Table 2). Some differences among haplotypes are observed, which likely reflect both the complexity of the species’ ploidy and the presence of additional haplotigs in the larger assemblies. The first assembled haploid copy (hereafter hap1) was the most contiguous assembly characterized by 2,083 scaffolds, a total length of 1.94 Gbp, a contig N50 of 1.024 Mb, a scaffold N50 of 39.6Mbp, and a scaffold L90 of 276. Assembly statistics at each step of RagTag-based correction and scaffolding of hap1 are summarized in Supplementary Table S1. The assembly size was in agreement with the genome size estimated from HiFi reads and it was close to the 1.8 Gbp reported for *A*. *ruthenus* (Du et al. 2020). The 80% of the assembly was contained in the first 60 scaffolds, suggesting a close proximity to the chromosome scale for several scaffolds (Fig. 1d). Compared with the diploid chromosome-scale *A*. *ruthenus* assembly, our tetraploid assembly showed similar contiguity metrics at the scaffold level (Scaffold N50 39.6 vs 42.7 Mb) but starting from a more fragmented contig set (Contig N50 1.02 vs 8.8 Mb) as expected due to challenges posed by the sorting of polyploid genomes (Table 2).

**Table 2:** Genome assembly statistics for each assembled haploid copy of *Acipenser naccarii* and *Acipenser ruthenus* (GCF_902713425.1).

	hap1	hap2	hap3	hap4	*Acipenser ruthenus*
Total length (Gb)	1.94	1.74	1.65	1.84	1.89
Number of scaffolds	2,083	2,370	1,881	3,438	1,730
Scaffold N50 (Mb)	39.6	37.89	43.81	42.13	42.7
Scaffold L50	14	13	11	12	12
Scaffold N90 (Mb)	0.485	0.405	0.684	0.313	9.613
Scaffold L90	276	271	101	280	47
Number of contigs	4,788	5,872	5,744	8,291	2,163
Contigs N50 (Mb)	1.024	0.806	0.756	0.700	8.8
Contigs L50	512	555	581	653	65

The hap1 assembly was moderately complete, reaching a BUSCO score of 88.9% according to the ctinopterygii_odb10 gene set and a k-mer completeness of 64.8% (Table 3). Both completeness statistics were significantly higher when computed jointly over the four assemblies, reaching a BUSCO score of 94.1% and a k-mer completeness of 95.2% (Table 3). The phred-scaled nucleotide error rate of the four assemblies was similar among them varying between 56.6 and 57.9, corresponding to one erroneous base every 450 and 620 Kbp, respectively. The estimated gene completeness was slightly lower than *A*. *ruthenus* genome (BUSCO 94.1% vs 98%) but still in line with the high-quality genome assembly standards (Rhie et al. 2021) (Table 3). To further assess assembly quality, we performed contamination screening using BlobTools for each haplotype, which did not reveal any evidence of obvious contamination (Supplementary Figs S3–S6). In addition, we evaluated k-mer completeness using Merqury, both by considering all haplotypes jointly (Supplementary Fig. S7) and each haplotype separately (Supplementary Fig. S8). These analyses indicate a high level of k-mer completeness with little missing single-copy genome content, whereas the observed duplicated k-mers are consistent with the presence of haplotigs resulting from haplotype-resolved assembly, particularly in haplotype 4, which shows a higher number of contigs and an increased proportion of duplicated BUSCO genes.

**Table 3:** BUSCO scores for each assembled haploid copy and all joint assemblies of *Acipenser naccarii* and *Acipenser ruthenus* (GCF_902713425.1).

	hap1	hap2	hap3	hap4	Joint assemblies	*Acipenser ruthenus*
Complete BUSCO (%)	88.9	88.7	87.4	88.1	94.1	98
Complete and single-copy BUSCOs (%)	55.7	58.3	57.2	51.5	2.3	27.4
Complete and duplicated BUSCOs (%)	33.2	30.4	30.2	36.6	91.8	70.6
Fragmented BUSCOs (%)	2.3	2.3	2.2	2.6	1.9	0.2
Missing assembly BUSCOs (%)	8.8	9	10.4	9.3	4	1.8
k-mer completeness (%)	64.8	63.6	63.2	65.5	95.2	/
QV	57.9	57.5	56.9	56.6	57.2	49.83

We applied nQuire to the first 60 scaffolds of the *A. naccarii* hap1 assembly individually. Thirty-four scaffolds fit the tetraploid model, whereas 13 matched the diploid model and two the triploid model (Supplementary Fig. S9). The remaining scaffolds did not conform to any tested model, potentially reflecting genomic regions with higher ploidy or issues related to model inference, as suggested by Smudgeplot analyses.

The mitochondrial genome was 17,033 bp with successful annotations of all 37 genes found in the reference mitochondrial genome (Supplementary Fig. S10).

### Genome annotation

We generated 16 Gb of strand-specific RNA-seq data to support genome annotation. A range of 27,050 to 29,814 protein-coding genes (36,458 to 40,061 transcripts) were predicted across the four haploid *A. naccarii* genome assemblies (Supplementary Table S2). The four annotations captured between 90.7% and 92.2% of the BUSCO Actinopterygii gene set as complete copies (Supplementary Table S2). The identification of repetitive elements in the four haplotypes revealed a repeat content ranging from 40.67% to 45.96% (Supplementary Tables S3–S6). The major class of repetitive elements was constituted by DNA transposons, with the DNA_hAT family representing the most abundant component, accounting for 11.56% to 14.58% of the genome across the haplotypes (Supplementary Fig. S11). Given their abundance, DNA_hAT elements may have contributed to ancestral genome duplications and structural evolution in sturgeons, representing an interesting focus for future study.

### Synteny

**Fig. 2:**
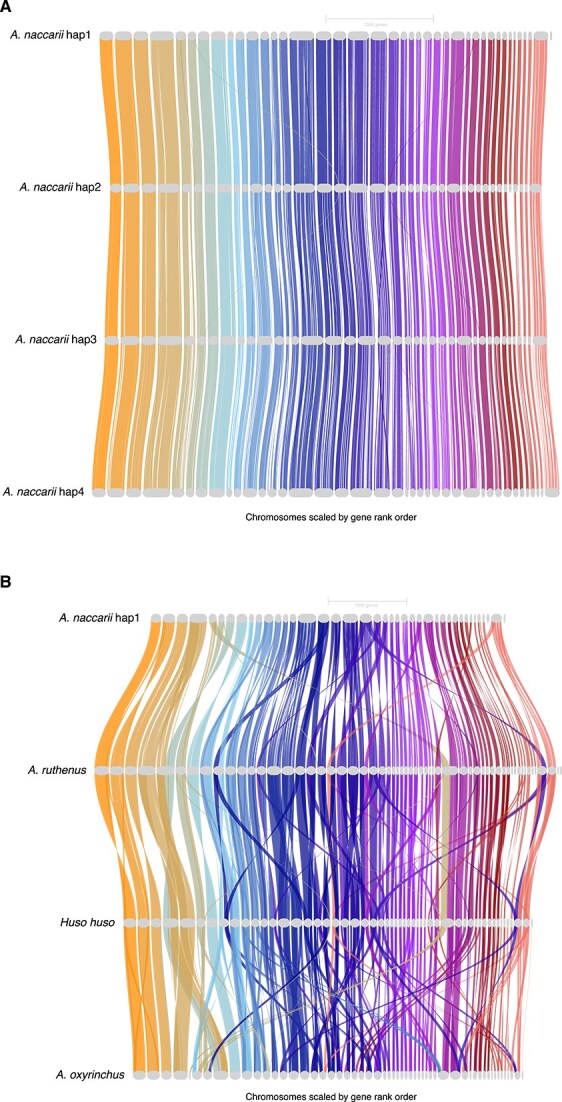
Synteny among the four *Acipenser naccarii* haploid assemblies and compared with *Acipenser ruthenus*. GENESPACE generated synteny map among (bottom to top) the four genome assemblies of *A. naccarii* (a) and among the *A. naccarii* hap1, *A. ruthenus*, *Huso huso*, and *Acipenser oxyrinchus* (b). Chromosomes are shown as segments, and syntenic blocks between each pair of genomes are shown as connecting links. Only scaffolds with strong and unambiguous syntenic signal were included.

The comparison of the four haploid *A. naccarii* genome assemblies revealed a high degree of synteny (Fig. 2a), confirming the consistency of all copies within our assembly (for details of the order across haplotypes, see Supplementary Table S7).

Additionally, a comparative analysis between the genomes of *A. naccarii* (hap1) and *A*. *ruthenus* demonstrated a high level of collinearity and synteny, with the majority of chromosomes showing no signs of major structural rearrangements (Fig. 2b). Although a few rearrangements were detected, their presence is expected given that these two species have been evolving separately for approximately 11.2 million years (Kumar et al. 2017). Such variations likely reflect natural evolutionary divergence rather than errors in genome assembly. It should be noted that the apparent difference in size between the assemblies in the synteny plots reflects that only orthologous genes shared across genomes are shown.

When comparing *A. naccarii* to *H. huso* and *A. oxyrinchus*, the extent of chromosomal rearrangements increased, particularly in *A. oxyrinchus*, which diverged from the other species approximately 171 million years ago (Peng et al. 2007) (Fig. 2b). This greater divergence is consistent with the accumulation of structural variations over the evolutionary timescale and highlights the deep phylogenetic split between *A. oxyrinchus* and the other sturgeon species. It is worth noting that the classification of *H. huso* in a genus different from *Acipenser* is not supported by molecular data and that this species is actually grouped together with *A. naccarii* within the cluster of Atlantic-origin species, whereas *A. oxyrinchus*, along with *Acipenser sturio*, forms the basal cluster of all sturgeon species. (Krieger et al. 2008).

## Conclusions

We present the first high-quality genome assembly for the Adriatic sturgeon, *A. naccarii*, a critically endangered, tetraploid species. Despite the challenges of assembling polyploid genomes, our phased assembly achieved high levels of completeness and contiguity, comparable to those of other recently assembled sturgeon species. We found a high degree of synteny among the haplotypes and with the closely related *A*. *ruthenus*. In contrast, deeper genomic divergence was observed in *A. oxyrinchus*, reflecting long-term structural rearrangements. Our analysis revealed a predominantly tetraploid genome with complex patterns of ploidy variation, offering new insights into genome evolution in sturgeons following whole-genome duplication events. The integration of genome assembly and annotation with transcriptomic data presents a valuable opportunity to investigate the process of functional diploidization and its impact on gene regulation, expression patterns, and evolutionary trajectories. In particular, the identification of heterozygous regions and allele-specific expression patterns will enable detailed studies of trait segregation and inheritance. This approach not only aids in understanding the evolutionary dynamics of tetraploidy but also validates candidate genes associated with diploidization, providing critical insights into the biology and adaptive potential of sturgeons. This genomic resource also has significant practical implications. It facilitates the construction of specific genetic markers, such as MIPs (Boscari et al. 2024), in order to support the development of robust conservation strategies, including population genetic studies, and the identification of breeding lines with optimal genetic diversity. In conclusion, this genome enhances our understanding of polyploid evolution in vertebrates and provides a critical resource for the conservation of this ancient and endangered species.

## Supplementary Material

SupFigs_Acipenser_naccarii_genome_revised_18Jan26_esag023

SupTabs_Acipenser_naccarii_genome_revised_18Jan26_esag023

## Data Availability

Raw sequencing data and genome assemblies are available under NCBI BioProjects PRJNA1259054, PRJNA1267827, PRJNA1267829, PRJNA1267830, PRJNA1267831. Genome assemblies, genome annotations, and RepeatMasker outputs are available on Zenodo (10.5281/zenodo.15705491). The mitochondrial genome sequence is available in GenBank under accession number PX733385.1 Scripts for removing multiple allelic haplotigs from genome assemblies are available at https://github.com/POPGG-UniFe/Sturgeon_genome.
