# Supplementary material for: Chromosome-level genome of the Adriatic sturgeon, Acipenser naccarii: A resource for polyploid fish genomics
Source: J Hered. 2026 Mar 12;117(4):877–87. doi: 10.1093/jhered/esag023 (PMC13326416; doi:10.1093/jhered/esag023)
Supplement: SupFigs_Acipenser_naccarii_genome_revised_18Jan26_esag023 [file supfigs_acipenser_naccarii_genome_revised_18jan26_esag023.docx]

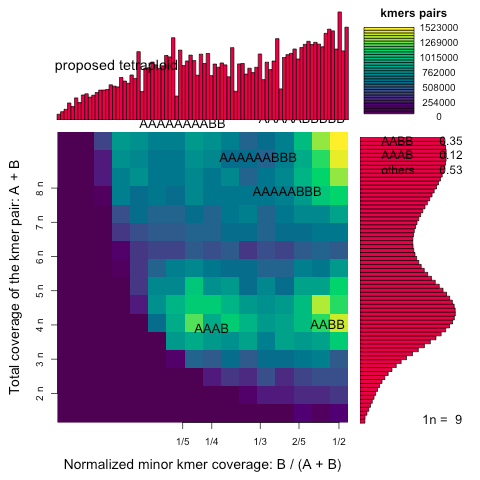


**Supplementary Fig. 1.** Ploidy composition of *Acipenser naccarii*. The letters A and B in the Smudgeplot represent a pair of heterozygous k-mers with only one SNP difference. The brightness of each smudge is determined by the number of heterozygous k-mer pairs that fall within it.


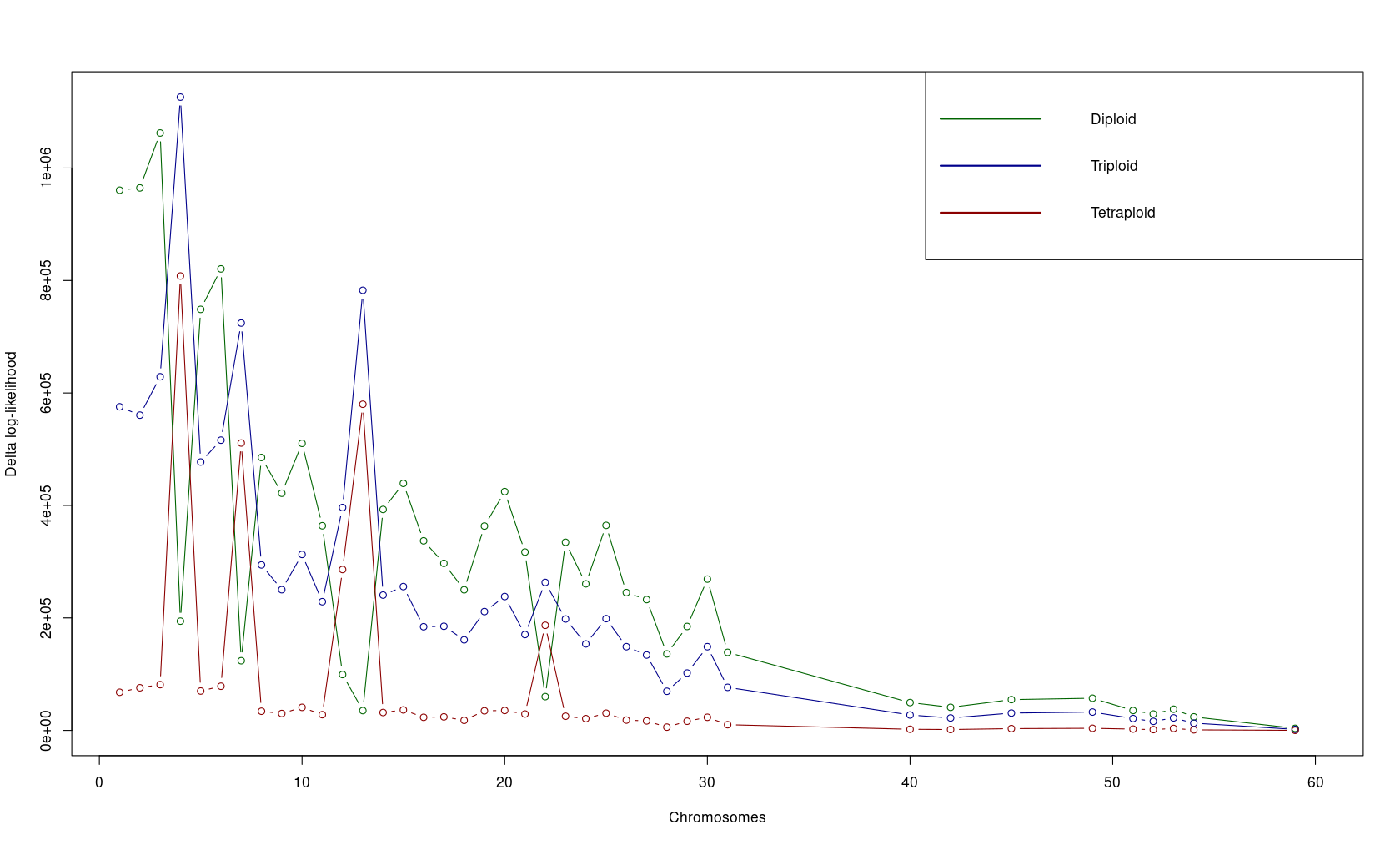


**Supplementary Fig. 2.** Ploidy estimation using nQuire for each of the 60 chromosomes of *Acipenser ruthenus*. The ΔlogL values (y axis) show a fit to the model (diploid, triploid or tetraploid). Chromosomes without dots did not fit any model.

**
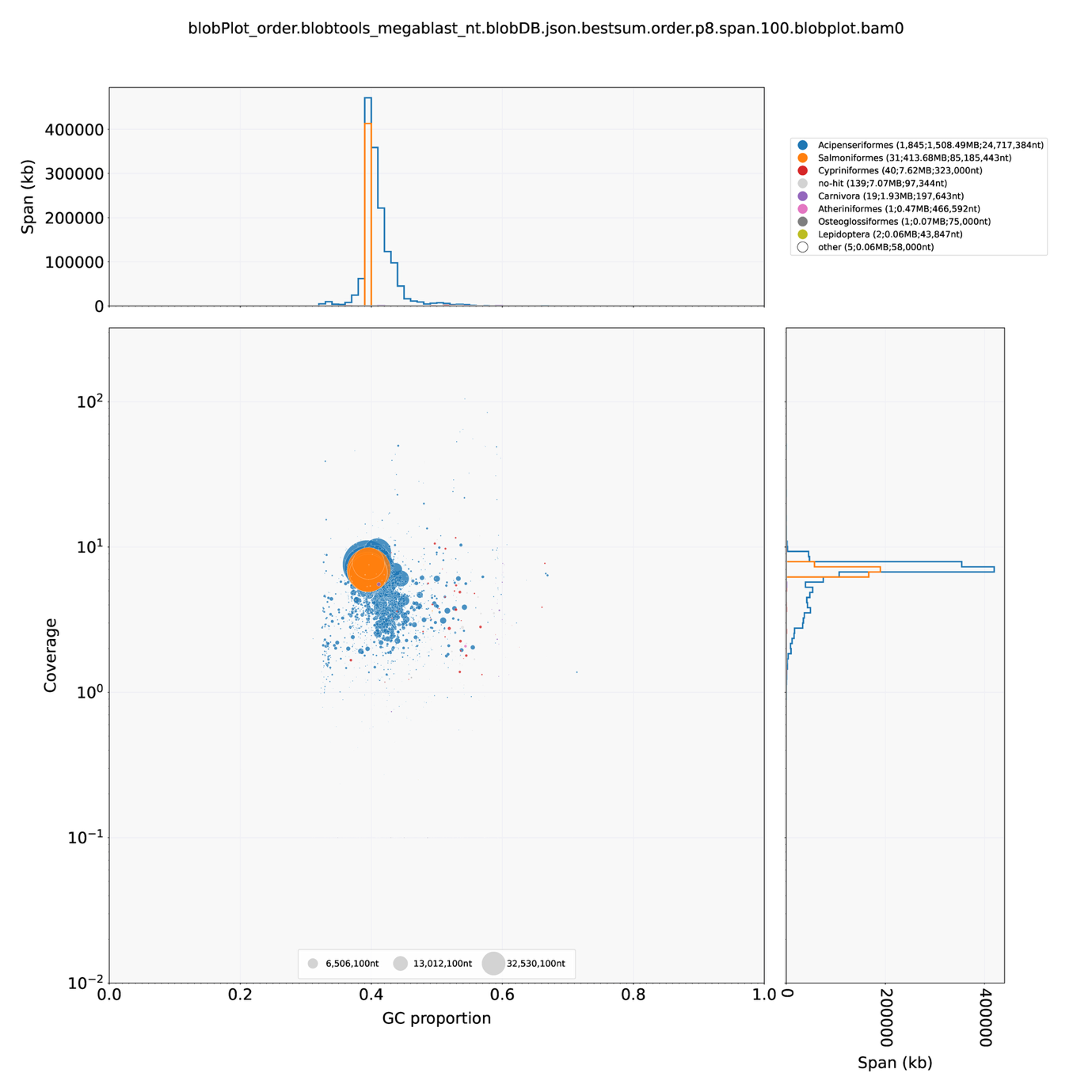
**

**Supplementary Fig. 3.** Taxon-annotated GC content–coverage plot of the hap1 genome assembly. Each circle represents a scaffold, scaled by length and colored according to order-level NCBI taxonomy assigned by BlobTools. The X-axis shows the average GC content of each scaffold, and the Y-axis shows the average coverage based on alignment of raw reads. Marginal histograms display cumulative genome content (in Kb) for bins of GC content (X-axis) and coverage (Y-axis).

**
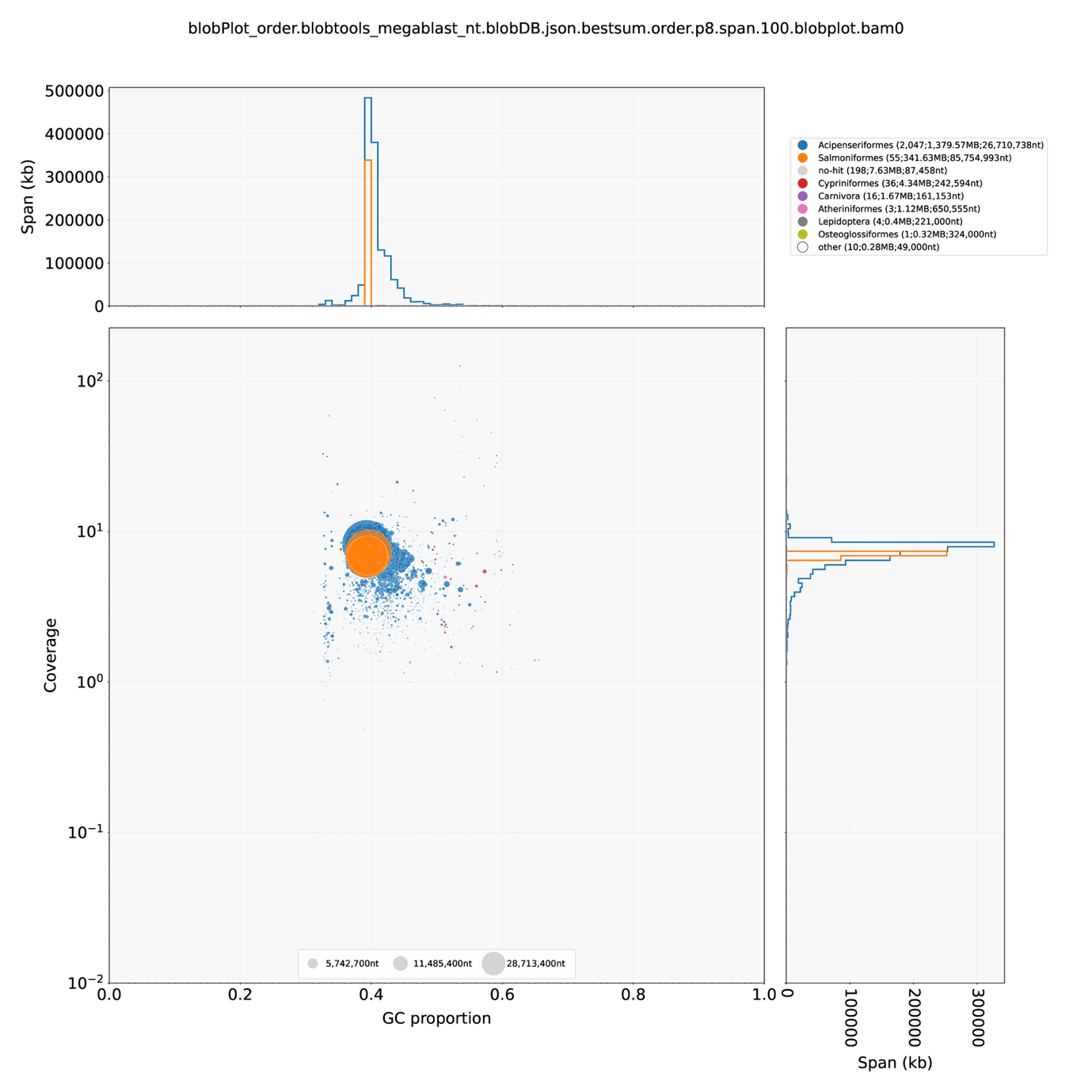
**

**Supplementary Fig. 4.** Taxon-annotated GC content–coverage plot of the hap2 genome assembly. Each circle represents a scaffold, scaled by length and colored according to order-level NCBI taxonomy assigned by BlobTools. The X-axis shows the average GC content of each scaffold, and the Y-axis shows the average coverage based on alignment of raw reads. Marginal histograms display cumulative genome content (in Kb) for bins of GC content (X-axis) and coverage (Y-axis).

**
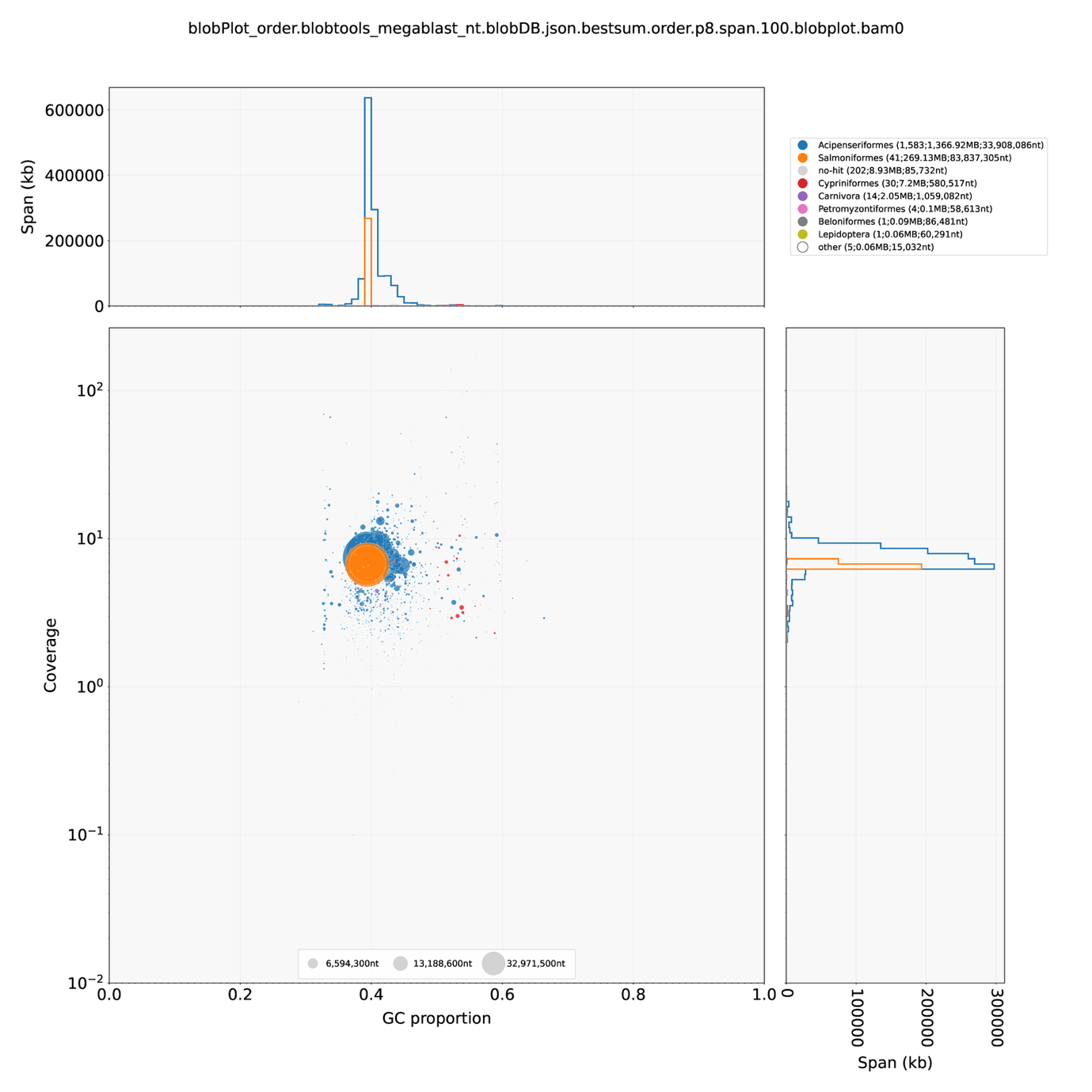
**

**Supplementary Fig. 5.** Taxon-annotated GC content–coverage plot of the hap3 genome assembly. Each circle represents a scaffold, scaled by length and colored according to order-level NCBI taxonomy assigned by BlobTools. The X-axis shows the average GC content of each scaffold, and the Y-axis shows the average coverage based on alignment of raw reads. Marginal histograms display cumulative genome content (in Kb) for bins of GC content (X-axis) and coverage (Y-axis).

**
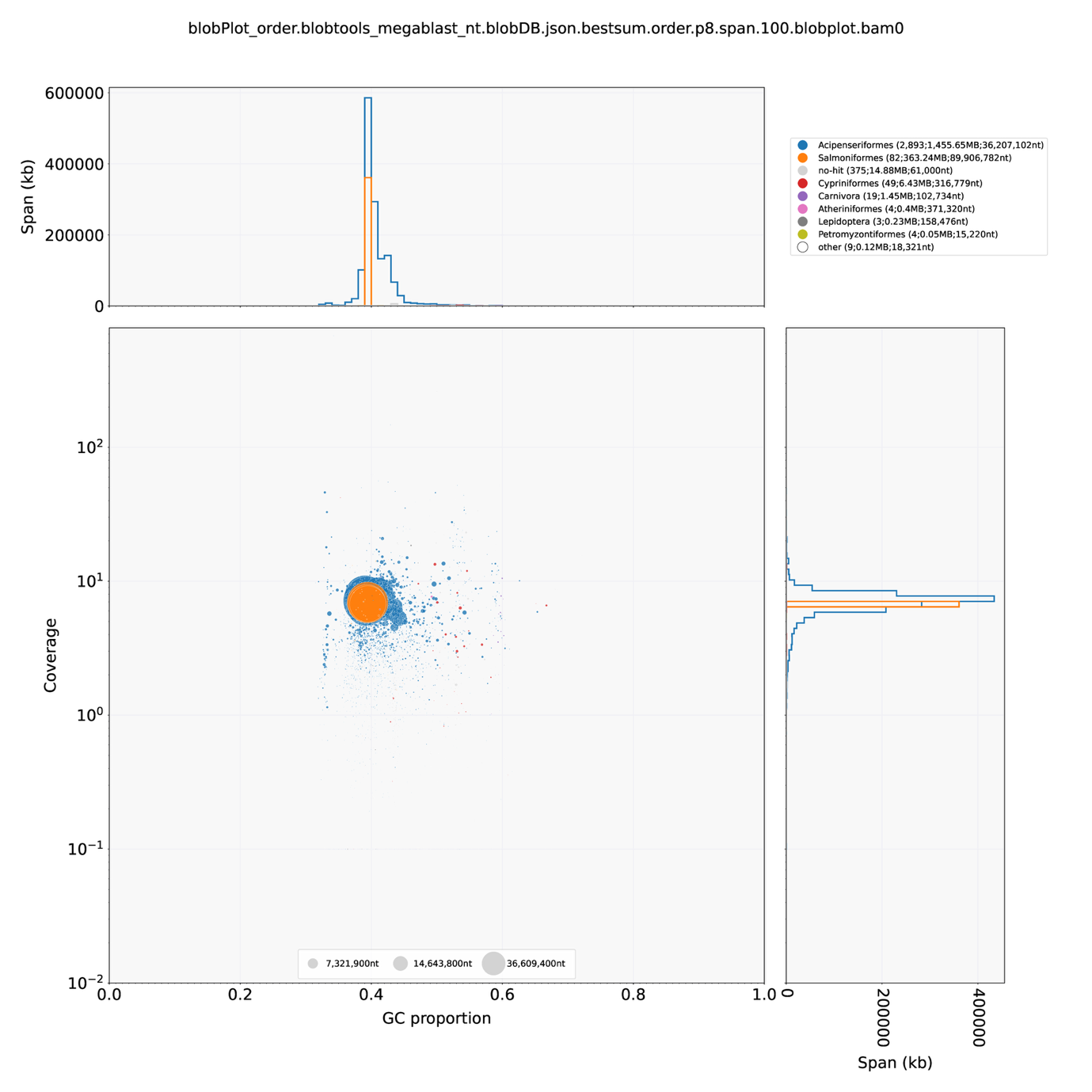
**

**Supplementary Fig. 6.** Taxon-annotated GC content–coverage plot of the hap4 genome assembly. Each circle represents a scaffold, scaled by length and colored according to order-level NCBI taxonomy assigned by BlobTools. The X-axis shows the average GC content of each scaffold, and the Y-axis shows the average coverage based on alignment of raw reads. Marginal histograms display cumulative genome content (in Kb) for bins of GC content (X-axis) and coverage (Y-axis).

**Supplementary Fig. 7.** Merqury k-mer completeness plot for all four haplotypes combined. The black area represents k-mers present in the reads but absent from the assembly, the red area represents k-mers present in the reads and occurring once in the assembly, and other colors indicate k-mers found multiple times in the assembly.

**Supplementary Fig. 8.** Merqury k-mer completeness plots for each haplotype. The four panels show the k-mer spectra for hap1 (A), hap2 (B), hap3 (C) and hap4 (D). In each plot, the black area represents k-mers present in the reads but absent from the assembly, the red area represents k-mers present in the reads and occurring once in the assembly, and other colors indicate k-mers found multiple times in the assembly.

**
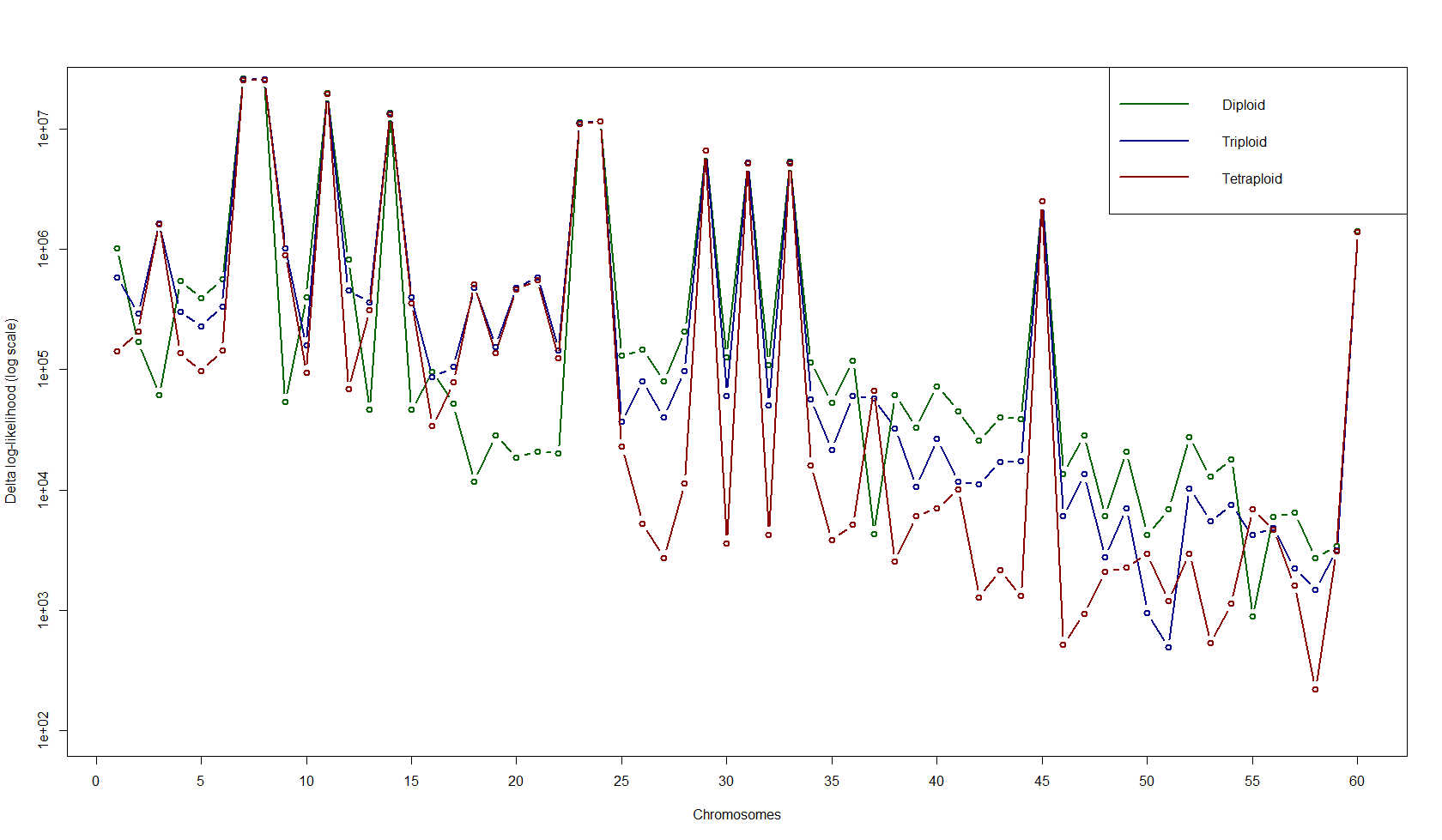
**

**Supplementary Fig. 9.** Ploidy estimation using nQuire for the 60 longest scaffolds of the *Acipenser naccarii* genome. The ΔlogL values (y axis) show a fit to the model (diploid, triploid or tetraploid).

**
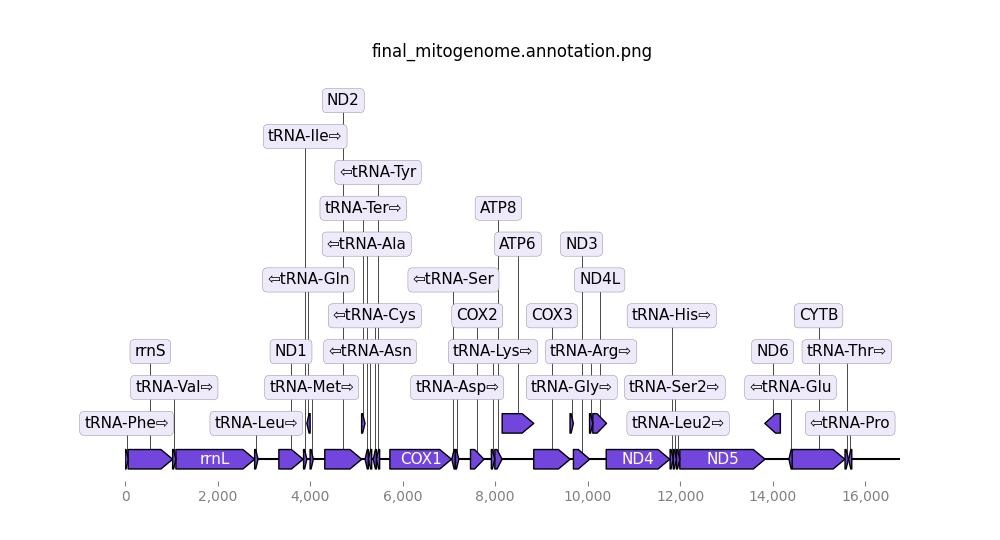
**

**Supplementary Fig. 10.** Mitochondrial genome annotation generated with MitoHiFi.


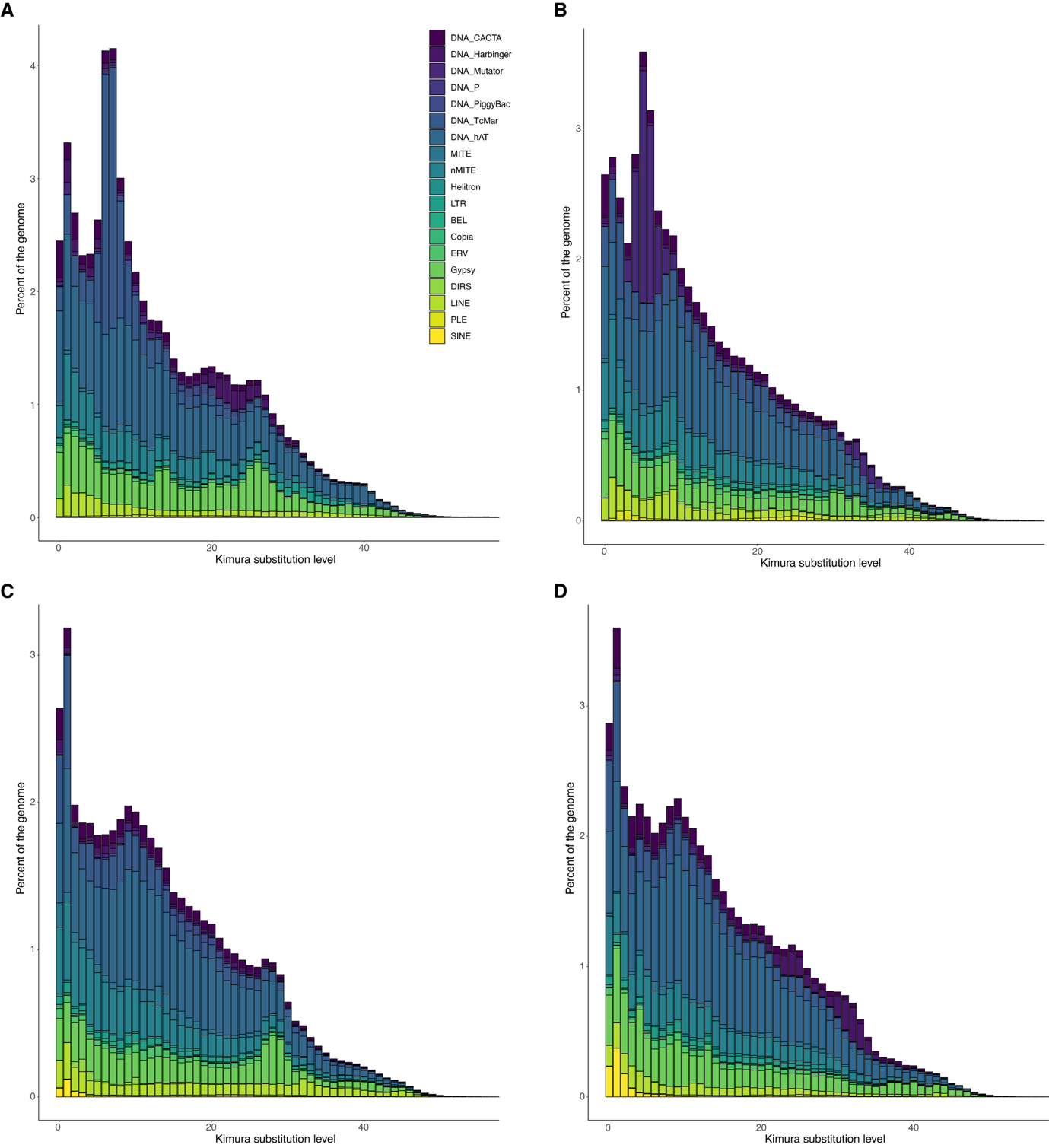


**Supplementary Fig. 11.** TE landscape plots obtained using Kimura distance-based copy divergence analyses in *Acipenser naccarii* for hap1 (A), hap2 (B), hap3 (C) and hap4 (D). X axis: Kimura substitution level; Y axis: percent of genome.
